# The Evolution of the Classification of Psychiatric Disorders

**DOI:** 10.3390/bs6010005

**Published:** 2016-01-18

**Authors:** Alina Surís, Ryan Holliday, Carol S. North

**Affiliations:** 1 Veterans Affairs North Texas Health Care System, 4500 S. Lancaster Road, Dallas, TX 75216, USA; ryan.holliday@utsouthwestern.edu; 2 University of Texas Southwestern Medical Center, 5323 Harry Hines Blvd., Dallas, TX 75390, USA; carol.north@utsouthwestern.edu; 3 Metrocare Services, 1380 Riverbend Dr., Dallas, TX 75247, USA

**Keywords:** DSM, psychiatric diagnosis, diagnostic classification, nosology, history of diagnosis

## Abstract

This article traces the history of classification systems for mental illness and then reviews the history of the American diagnostic system for mental disorders. The steps leading up to each publication of the Diagnostic and Statistical Manual (DSM) are described including leaders, timelines, pre-publication meetings, and field trials. Important changes in the purpose of the manuals are described with a focus on events leading to the manual’s third edition (DSM-III), which represented a paradigm shift in how we think about, and use, the classification system for mental illness. For the first time, DSM-III emphasized empirically-based, atheoretical and agnostic diagnostic criteria. New criticisms of the DSM-III and subsequent editions have arisen with a call for a new paradigm shift to replace diagnostic categories with continuous dimensional systems of classification, returning to etiologically-based definitions and incorporating findings from neurobiological science into systems of diagnosis. In the foreseeable future, however, psychiatric diagnosis must continue to be accomplished by taking a history and assessing the currently established criteria. This is necessary for communication about diseases and education of clinicians and scientists in medical fields, as well as advancement of research needed to further advance the diagnostic criteria of psychiatry.

## 1. Introduction

Diagnosis is of vital importance to everything that is done in medicine: it is critical to applying effective and appropriate treatment, it informs the patient’s prognosis, it enables communication about diseases among clinicians and scientists, it is foundational to medical education, and it is necessary for the conduct of research [1,2,3]. Reliable diagnosis is also needed for accurate estimates of prevalence rates of disorders, health service planning, and documenting vital public health information such as morbidity and mortality sequelae of disease [4]. Before organized systems of diagnostic classification were developed, the field of medicine had no formal basis to support the validation of its practices, a situation that did little to improve the limited public respect for the field in previous eras [5]. The development of systems for classification of medical diseases was critical to the development of the field of medicine and the advancement of medical science.

Classification of medical illness inherently involves principles of nosology, or the science of classification of diseases. Disease has been generally defined by medical experts as:

An impairment of the normal state of the living animal or plant body or one of its parts that interrupts or modifies the performance of the vital functions, is typically manifested by distinguishing signs and symptoms, and is a response to environmental factors (as malnutrition, industrial hazards, or climate), to specific infective agents (as worms, bacteria, or viruses), to inherent defects of the organism (as genetic anomalies), or to combinations of these factors: sickness, illness—called also morbus [6].

Mental disorders are considered medical illnesses [3]. Mental disorder was consistently defined in the American Psychiatric Association’s Diagnostic and Statistical Manual of Mental Disorders, Third Edition (DSM-III) [7], Third Edition-Revised (DSM-III-R) [8], and Fourth Edition (DSM-IV-TR) [9] as:

A clinically significant behavioral or psychological syndrome or pattern that occurs in an individual and that is associated with present distress (e.g., a painful symptom) or disability (*i.e.*, impairment in one or more important areas of functioning) or with a significantly increased risk of suffering death, pain, disability, or an important loss of freedom. In addition, this syndrome or pattern must not be merely an expectable and culturally sanctioned response to a particular event, for example, the death of a loved one [10].

Physicians have long observed that medical afflictions tend to fall into syndromes with relatively stable patterns of signs and symptoms. Identifying such syndromes has considerable utility for medical practice, education, and research. Among patients who have similar signs and symptoms, the prognosis might be expected to be similar. Additionally, underlying causes could be identified by finding commonalities in the histories of different patients with the same signs and symptoms. Finally, treatments found to help one patient might be tried for other patients with the same signs and symptoms [11]. Medical diagnosis and the ability to differentiate disorders from one another is the foundation of clinical practice. Choosing the most effective treatment depends, for example, on whether the patient has pneumonia, pulmonary embolus, congestive heart failure, or lung cancer. These different conditions, although they affect the same parts of the body and share some features of their presentation, require different treatments. Differentiating among various disorders is equally important in psychiatry as in the rest of medicine. For example, schizophrenia, mood disorders, and substance use disorders are different illnesses that have been demonstrated to have different prognoses and require different treatments [2,3].

## 2. Early History of Classification Systems for Mental Illness

By the late 1800s, medical science was making great advances in the understanding of the biological origins of medical illness, especially with the discovery of bacteria as the source of infectious diseases. In that era, German physicians Kraepelin and Alzheimer were developing methods to identify neurological causes of disease in some of their patients and to separate diseases such as dementia from other psychiatric illnesses on the basis of biological indicators. They further advanced the notion that mental illness may have a biological basis, and began to organize a framework of psychiatric disorders based on systematic observation of patterns of illness, including characteristic symptoms, course, and outcomes among patients. Their work, however, was largely ignored by American psychiatry at the time [12].

It was not until 1844 that psychiatry was first recognized as a medical specialty in the United States, by the Association of Medical Superintendents of American Institutions for the Insane, an organization that became the American Psychiatric Association (APA) in 1921. At that time, American institutions all had their own in-house diagnostic systems, generally based on prototypical case studies with application of diagnoses to cases based on consensus of patients’ treating physicians. In about 1940, American psychiatry came to be dominated by psychoanalytic theory, an era that lasted approximately two decades [13]. During this period, American psychiatry emphasized individual differences rather than commonalities in illnesses. Mental processes in psychological health and illness were assumed to be similar. The lack of a unified classification system and the lack of progress toward embracing a biological appreciation of psychiatric illness led to the marginalization of American psychiatry from the rest of medicine [12]. Psychoanalysis eventually came under attack in American psychiatry with two developments. The first was the discovery of psychiatric medications that were increasingly used for the treatment of major psychiatric illness. The second was the advent of biological research into mental disorders with important new discoveries such as neurotransmitter systems [14].

The first American initiative to develop standardized diagnostic criteria was prompted by the U.S. Census Bureau, to aid efforts to estimate the prevalence of mental disorders in America for the 1920 census. This initiative produced a diagnostic manual, the Statistical Manual for the Use of Institutions for the Insane (SMUII), which outlined 21 disorders, 19 of which were psychotic disorders. This manual was largely ignored by American psychiatrists, even through the evolution of this document across a series of 10 editions by 1942 [12]. In 1952 and 1968, the American Psychiatric Association released its first two versions of its diagnostic criteria for psychiatric disorders, but diagnostic reliability and validity were not to be established until decades later.

In 1970, the classic US–UK cross-national study illustrated the importance of having a unified diagnostic system for determining rates of psychiatric illness. Gurland and colleagues [15] conducted a study to clarify large reported discrepancies between U.S. (New York City) and UK (London) statistics on the proportions of adults with hospital admissions for schizophrenia and manic-depressive illness. Utilizing semi-structured interviews, ratings of videotapes, and systematic examination of case records, the study found that inconsistent diagnostic methods for routine hospital admissions between the sites were responsible for large discrepancies in diagnosis. Many of the patients diagnosed with schizophrenia in New York would have been diagnosed with manic-depressive illness in London. Diagnostic agreement would not be possible until reliable diagnostic systems came into existence, such as with DSM-III [7] in 1980.

## 3. A Paradigm Shift in the Conceptualization of Diagnostic Criteria for Mental Disorders

In the mid-20th century, a revolution was beginning in St. Louis, Missouri which would ultimately transform American psychiatry. Eli Robins, Samuel Guze, and a small group of researcher/clinicians in the “Renard School” of psychiatry at Washington University, eschewed the dominant practice in American psychiatry of psychoanalysis, which was theoretically and etiologically based and inherently opposed to psychiatric diagnosis [13]. These academicians were dissatisfied with existing methods of diagnostic classification in psychiatry, which were based more on clinical opinion than on systematic research [1]. Believing that reliable and valid diagnostic criteria were essential for the field of psychiatry to establish meaningful treatments and conduct scientific research [1,2], this pioneering group set an ambitious goal of developing operationalized diagnostic criteria. In this work, they specifically avoided theoretical assumptions about the etiology of psychiatric illness in an atheoretical and etiologically agnostic approach to defining psychiatric disorders [3]. Broader than simply defining disease, this work encompassed strategies for thinking about, studying, and providing care for patients with psychiatric illness. The St. Louis approach to psychiatric diagnosis came to be known as the “medical model” of psychiatry, which incorporated psychological and social contexts along with biological aspects of psychiatric illness [3].

In developing diagnostic criteria for psychiatric disorders, Robins and Guze emphasized the importance of validity (whether a coherent syndrome is being measured, and whether it is what it is assumed to be) as well reliability (the likelihood that different clinicians arrive at the same diagnosis). To address validity, they adopted a well-established five-phase diagnostic validation model for operationalization of criteria for medical diagnosis. This validation method dates back to work in the 17th century by Thomas Sydenham, and further includes contributions of Koch, Pasteur, and Virchow in the centuries to follow [1,13]. Its application to psychiatric diagnosis in the 20th century, however, was considered radical at the time [16]. The five phases of diagnostic validation used by Robins and Guze were: (1) clinical characteristics of the syndrome and of the patients who develop it (including core symptoms, demographic characteristics, and precipitating factors); (2) exclusionary criteria differentiating the syndrome from other known disorders; (3) family studies; (4) laboratory data (radiological, chemical, pathologic, and psychological evidence); and (5) follow-up studies (for diagnostic stability, course, and treatment response).

The Robins and Guze validation procedure is considered a gold standard for judging different sets of criteria for diagnostic categories. The validation process was intended to be iterative, and adjustments based on the availability of new data to further improve the criteria would always be possible [1,17]. Robins and Guze noted that psychiatric science generally lacked biological evidence, and that more complete diagnostic validation procedures incorporating the fourth phase of validation (laboratory data) thus awaits future advances in biological science [1].

Kendler and colleagues [13] identified three major contributions of the Robins/Guze methods: (1) systematic application of operationalized criteria to psychiatric diagnosis; (2) a basis in empirical data rather than clinical opinion to optimize diagnostic criteria; (3) emphasis on course and outcome as a critical defining feature of psychiatric illness. The course and outcome to define psychiatric categories of illness related to this third contribution of Robins and Guze had actually already come into use in 19th century Europe, as illustrated by the work of Kraepelin in distinguishing schizophrenia from manic-depressive illness based on its characteristic time course [13]. The influence of the work of Kraepelin on their methods prompted the use of the term “neo-Kraepelinian” to refer to the Washington University School of Psychiatry [16].

Defining diagnoses descriptively and empirically based on characteristic symptoms and course of illness, rather than on theoretically based in assumed etiologies, has practical implications [3]. Scientifically untested causal assumptions about the etiology of disease may be incorrect, leading to misguided treatment. An agnostic approach to diagnosis not assuming etiology opens doors to testing of causal hypotheses through epidemiologic, genetic, and neurobiological research.

In the 1970s, Feighner, Robins, Guze, Woodruff, Winokur, and Muñoz [17] operationalized and demonstrated validity for a set of specific diagnostic criteria for adult psychiatric disorders for use in both research and clinical practice. These criteria became known as “the Feighner criteria.” The Feighner criteria addressed diagnoses of primary affective disorders (depression and mania) and secondary affective disorder (depression only), schizophrenia, anxiety neurosis, obsessive-compulsive neurosis, phobic neurosis, hysteria, antisocial personality disorder, alcoholism, drug dependence, mental retardation, and anorexia nervosa. The criteria also included an entity for “undiagnosed psychiatric illness”, providing a diagnostic option for psychiatric disorders that, for one or more of a several potential reasons, cannot be determined to fit any other specific diagnosis—which Guze and colleagues found applicable to as many as one third of their cases [3].

Helzer and colleagues [18] pointed out that valid diagnostic criteria are of little use if clinicians cannot consistently agree about a diagnosis attained through application of the criteria, as determined through inter-rater reliability. The development and measurement of reliability using validated diagnostic criteria, however, would have to await the advent of structured interview methods, which were developed over the next few years and used to document acceptable reliability of established diagnostic criteria [19,20,21,22].

## 4. The American System of Diagnostic Criteria

By the 1950s, five separate “official” diagnostic classification systems were being used in the United States in different settings, including the insane asylum system, the Army, the Navy, the Department of Veterans Affairs (VA), and the American Prison Association [12]. The American Psychiatric Association set out to create a unified and definitive diagnostic system for all of American psychiatry. In 1952, the Diagnostic and Statistical Manual, Mental Disorders (DSM) was published [23]. This diagnostic system was based on the Veterans Administration (VA) system of psychiatric diagnosis. Drafts of newly proposed criteria were circulated to 520 American and Canadian psychiatrists, and 241 replies were received. The manual was divided into two main sections, one for disorders with established organic brain disease and the other for disorders without evidence of organic brain findings. The latter disorders were labeled “functional” and were subdivided into disorders of psychosis, psychoneurosis, and personality. The classification of psychiatric disorders in the 1952 DSM was etiologically based; the nomenclature of the mental disorders as “reactions” to stressors (e.g., “depressive reaction” and “schizophrenic reaction”) clearly implied assumptions of psychodynamic causality [12].

The American Psychiatric Association published a second edition of the Diagnostic and Statistical Manual of Mental Disorders (DSM-II) in 1968 [24]. This revision of the original DSM was prompted by a desire to increase the compatibility of the American and international diagnostic systems and address inconsistencies in criteria between the DSM and the World Health Organization’s International Classification of Diseases (ICD) that was in its 8th edition at the time [12]. DSM-II expanded the number of diagnostic sections from two to ten and it added a child/adolescent section. Because the first DSM was designed to generate population statistics, it did not permit diagnostic comorbidities. DSM-II reversed the original manual’s prohibition against diagnostic comorbidity. It retained the psychodynamic nomenclature and etiologically-based classification of the first DSM, but the term “reaction” was removed, possibly representing an initial step toward an atheoretical etiologic orientation in future versions of the diagnostic criteria.

In the 1970s, Robert Spitzer of the New York State Psychiatric Institute was selected to head the revision of American diagnostic criteria for DSM-III. This was timely, because Spitzer had previously been funded by the National Institute of Mental Health to develop a new set of diagnostic research criteria, leading him to consult with Eli Robins, senior mentor of the Feighner criteria team and chairman of psychiatry at Washington University. This group used the Feighner criteria as a blueprint for the creation of Spitzer’s new Research Diagnostic Criteria (RDC), with an expanded number of diagnoses [25]. Based on the Feighner and RDC criteria, Spitzer introduced the formal operationalization of psychiatric diagnosis with established reliability and validity into DSM-III and provided a new hierarchical, multi-axial system for diagnosis utilizing exclusion criteria. The DSM-III replaced psychodynamic formulations and related terminology with criteria that were atheoretical and agnostic with regard to etiology of psychiatric disorders [26]. As a concession to psychodynamic practitioners, the term “neurosis” was retained in the nomenclature, but only as a parenthetical note, and it was removed from future DSM editions. As with DSM-II, the plan was to make DSM criteria compatible with ICD criteria. In a surprising reversal, the new DSM criteria were found to be so far superior to the ICD criteria that the ICD-9 criteria were modified to be consistent with DSM-III, rather than the original plan to mold the DSM criteria to fit ICD criteria [12].

The changes in the diagnostic criteria in DSM-III were highly controversial and contributed to a radical redirection of American diagnostic criteria for psychiatric disorders that represented a major paradigm shift [26,27,28]. These advancements were part of a larger movement at the time in American psychiatry to re-medicalize psychiatry, grounding the field in empirical research. The new empirically-based and operationalized definition of mental disorders in DSM-III added legitimization to the field as a medical specialty [12,27].

Spitzer was again selected to head the creation of the next edition of the criteria, and DSM-III-R was published in 1987 [12]. Intended as a revision to the previous criteria, DSM-III-R further refined the criteria for utilitarian value based on suggestions from practicing clinicians and researchers [12]. The diagnostic hierarchy was removed, leading to sharply increased comorbidity findings in epidemiological research studies to follow.

Only one year after publication of DSM-III-R, a DSM-IV task force was appointed in 1988 utilizing Spitzer as an advisor. This time, Allen Frances, a psychoanalyst from New York who had worked on the personality disorders section of DSM-III, was chosen to head the next revision [12]. As with past editions of the criteria, development of the DSM-IV was prompted by the upcoming release of new international criteria (ICD-10) in 1993. DSM-IV was not published until 1994, but it had relatively few significant changes from the previous criteria [12]. Perhaps the most far-reaching change in DSM-IV was the systematic addition of “clinically significant distress or impairment” across the diagnostic criteria. A “text revision” version, DSM-IV-TR, was released in 2000 for the purpose of updating the research literature between 1992 and 1998. This edition of the criteria left the criteria essentially untouched, simply providing more detail in the accompanying text on associated features of disorders [12].

One year before the publication of DSM-IV-TR, planning commenced in 1999 for the next DSM edition. David Kupfer, a neuroscientist from Pittsburgh, was appointed as chair of the task force for DSM-V. The main priorities for the DSM-5 revision of the criteria were to incorporate etiological and neurobiological research into definitions of psychiatric disorders and to improve clinical utility of the criteria [29,30]. These objectives were supported by plans to incorporate dimensional and cross-cutting measures and developmental and environmental history into the diagnostic criteria and accompanying text descriptions [30,31]. A fundamental change to the DSM-5 psychiatric diagnosis system was the removal of the multi-axial approach to diagnosis. Rationale for removal of the multi-axial system, that was first introduced in DSM-III in 1980, was based on unclear boundaries between medical and psychiatric diagnoses, inconsistent use of Axis IV by clinicians and researchers (psychosocial and environmental problems), and poor psychometric and clinical validity of Axis V (Global Assessment of Functioning) [4,32,33,34,35]. When DSM-5 was finally released in 2013, the Roman numeral “V” in its name was replaced with the Arabic number “5” in a deliberate change of naming convention to create discontinuity from previous DSMs to emphasize a major departure in many conventions in the criteria. An additional advantage to this new naming convention was the facilitation of the naming of future revisions of the criteria (e.g., DSM-5.1, DSM-5.2, DSM-5.3, *etc.*) for infinite revisions within the fifth edition as frequently as needed [30].

The purpose of the diagnostic criteria has shifted over time. DSM-I and DSM-II were developed for the purpose of gathering statistical information on the prevalence of mental disorders [12]. The original purpose of the Feighner criteria that were used for DSM-III was to provide valid and reliable diagnosis for research [17], sorting patients into homogeneous samples needed for optimal signals in research data [2,36]. A main reason stated for the DSM-III-R criteria revision was to address clinical utility of diagnosis, and this goal was accomplished through input from clinicians [12]. In recent years, insurance companies, managed care organizations, pharmaceutical companies, and the government have increasingly utilized systematic diagnostic criteria for the reimbursement and financial aspects of clinical practice [27].

Across the editions of the DSM criteria, a number of disorders have come and/or gone. Some of these disorders deserve specific comment. Posttraumatic stress disorder and borderline personality disorder were not part of the American diagnostic criteria until first being included in DSM-III in 1980, and both of these diagnoses have persisted through the current edition of the criteria. Acute stress disorder, bipolar II disorder, and Asperger’s disorder did not exist until they were introduced into DSM-IV in 1994. In DSM-5, the independent diagnosis of Asperger’s disorder was removed and subsumed within autism spectrum disorder. One of the few diagnoses validated by Feighner and Robins and Guze *et al.* [17], somatization disorder (under its former name, hysteria), was represented in all of the editions of the DSM criteria until DSM-5, when it was removed and replaced with the new diagnosis of somatic symptom disorder. Diagnosis of homosexuality has a particularly complex history in the American diagnostic system. In DSM-I, homosexuality was classified with the sexual deviation disorders in the section on sociopathic personality disturbance, with retention of this diagnosis in DSM-II. In subsequent printings of the DSM-II beginning in 1973, the diagnosis of homosexuality was replaced with “sexual orientation disturbance.” The only reference to homosexuality in the DSM-III diagnostic criteria in 1980 was “ego dystonic homosexuality,” and by 1987, homosexuality was completely removed from the DSM-III-R diagnostic criteria [12].

An obvious change across the editions of the DSMs has been the increase in the amount of material in these manuals. The Feighner criteria that contributed to the original basis for the DSM-III criteria covered only about a dozen validated diagnoses and the RDC criteria modestly expanded the number of diagnosis. The number of diagnoses in DSM-III, however, climbed to 265 from the 106 diagnoses included in the first edition of the DSM criteria. This rapid growth in numbers of diagnoses slowed, however, yielding 292 diagnoses in DSM-III-R, 297 in DSM-IV and DSM-IV-TR, and 298 in DSM-5 [27]. The growth in numbers of diagnoses was also reflected in the manual’s volume, which began with 130 pages in the first edition and ballooned to 992 pages in DSM-5. A problem with the large numbers of psychiatric disorders in the formal system of diagnostic criteria is that validity and reliability historically established for a select group of psychiatric disorders has not been extended to most of the remaining diagnoses in the manual [37,38].

The length of time of task force deliberation to produce the DSMs has also increased over time. Both DSM-III and DSM-IV required six years of task force work; DSM-5 also took six years from the appointing of the task force, but the entire process of planning and preparing for DSM-5 spanned 14 years [12,30]. Another trend across successive editions of the DSMs has been a perceived increase in financial ties of its developers to the pharmaceutical industry, with the industry ties of the task force alone increasing from 57% to 72% between DSM-IV and DSM-5. Concerted efforts to increase transparency and limit members to monetary restrictions on pharmaceutical earnings in DSM-5 failed to eliminate perceptions of industry influence on the deliberations of its committees [39].

Field trials were conducted with each successive version of the DSMs beginning with DSM-III. The DSM-III, DSM-III-R, and DSM-IV field trials were conducted within one to three years before the publication of these editions, and the results were extensively used in the modification of the criteria for these editions of the manual in preparation for final publication. Field trials to measure reliability for the consideration of changes to diagnostic criteria were also conducted in the three years preceding the publication of DSM-5, but for only a very few select diagnoses of public health importance or for possible addition to the manual [4,36]. The DSM-III and DSM-IV field trials focused on reliability of the proposed criteria, and the DSM-IV trials included an additional focus on clinical utility and comparison of diagnostic prevalence based on criteria from DSM-III onward [36]. The DSM-5 field trials focused on feasibility and clinical utility of diagnoses and emphasized biological research not confined to diagnostic boundaries as currently conceptualized, consistent with the National Institute of Mental Health (NIMH) Research Domain Criteria (RDoC) project [36].

A criticism of the DSM-5 field trials was the low reliability obtained for the proposed DSM-5 diagnoses [40,41], e.g., the “questionable” level of intraclass kappa (0.28) of test-retest reliability for major depressive disorder [36]. However, the designers of the DSM-5 field trials were not seeking to obtain the levels of reliability found in the previous field trials, because, unlike the previous trials, the research was conducted with naturalistic patient samples from clinical settings and used non-expert clinicians to rate the diagnoses through checklists rather than with standard diagnostic interviews [36].

## 5. Conclusions

This article has reviewed the long history of diagnostic classification of psychiatric disorders. DSM-III heralded a paradigm shift in the history of psychiatric diagnosis, with its incorporation of empirically-based, atheoretical and agnostic criteria for psychiatric diagnosis [28]. With subsequent revisions of the diagnostic manual since DSM-III, however, increasing dissatisfaction with the validity of the criteria has become apparent with complaints that the criteria do not sufficiently differentiate disorders leading to high rates of diagnostic comorbidity, diagnosis lack specificity for selection of treatment, genetics fail to distinguish psychiatric disorders, and many observed syndromes do not fit any diagnostic definition [28].

Dissatisfactions with the current conceptualization of current diagnostic criteria in psychiatry have prompted calls for a new major paradigm shift [28,42]. Recommendations for changes in the diagnostic system with this paradigm shift have been made to replace diagnostic categories with continuous dimensional systems of classification; substitute etiologically-based definitions of disorders for the current descriptive and theoretically agnostic system; and incorporate findings from neurobiological science into systems of diagnosis [28,29,43]. Kendler and First [28] identified two strategies that could be followed in future efforts to revise psychiatric nosology: (1) an “iterative model” involving small incremental changes to the existing model; and (2) a “paradigm shift model” that discards the underlying paradigm to adopt a fundamentally new approach to diagnosis. These academicians concluded, however, that the field is not ready for a paradigm shift, because there is not a superior alternative paradigm that sufficiently addresses the identified shortcomings of the current system. Hyman [42] further concluded that the current progress in neurobiology is not yet adequate for it to contribute usefully to diagnostic classification.

Clinical diagnosis has not lost its utility over time. Utility of psychiatric diagnosis has increased, especially with recently increasing practical reliance on diagnosis in the form of billing codes for reimbursement for services rendered. However, the criteria for these diagnoses appear to be diminishing in utility in the application of diagnoses in practice in both clinical and research settings. Busy clinicians, faced with diminishing resources and increasing demands of their time, as exemplified by the “ten-minute med check”, often do not have the time needed to assess the criteria to fully justify the diagnoses they make. Researchers, needing to obtain large samples and reduce the burden of assessment in times of dwindling resources for research, are increasingly substituting self-report symptom measures for full diagnostic instruments. The end result is that the carefully considered and crafted criteria that have been established are not being used. Frustrations resulting from performance demands without sufficient resources have translated into desires for a biological test that can circumvent the time and effort needed for present methods of psychiatric diagnosis, but unfortunately this possibility is not yet available.

In the foreseeable future, we will continue to diagnose psychiatric disorders the old-fashioned way: by taking a history and assessing the currently accepted criteria to make diagnoses as they are provided in the established classification system. If we do not use the diagnostic criteria we have, despite the imperfections of existing criteria, the diagnostic system that exists will be wasted. Without accurate diagnosis, appropriate treatment cannot be selected, the prognosis cannot be known, communication about diseases among clinicians and scientists will flounder, education of clinicians and scientists in medical fields will suffer, and research will not advance—ironically, the very biologic research that the field needs to further advance the diagnostic criteria of psychiatry.
